# Some Diseases of the Male Genital System

**Published:** 1907-12-28

**Authors:** 


### SOME DISEASES OF THE MALE GENITAL SYSTEM.

### II.—THE DIAGNOSIS OF VAGINAL HYDROCELE.

Apart from the presence of a swelling in the scrotum, a pure hydrocele gives rise to no symptoms proper; and as its onset is insidious, it may have been present for some considerable time before the patient's attention is drawn to his condition by its increasing size. The diagnosis rests, therefore, more on the character of the swelling than on any information which can be obtained from the patient.

On examination one side of the scrotum will be found to be occupied by a smooth, pyriform mass which reaches up to the external abdominal ring, and is sharply limited by this. In a small percentage of cases the hydrocele is double, and it follows that a mass will be found on both sides. The testis is involved in, and moves with, the mass, and it may or may not be possible to locate it by palpation, or at any rate to produce the sensation of testicular pain by pressure in some given spot. Generally speaking, this can only be done if the hydrocele is fairly tense, so that the testis lies at some point of its periphery. A sensation of fluctuation may frequently be obtained; occasionally, however, though rarely, as in any other encysted collection of fluid in the body, the tension may be so great as to give the impression of a solid mass. Fortunately this rarely happens; when it does the pressure caused may evert an injurious action on the testis or the vasa efferentia, and lead to complete loss of function.

It does not give any impulse when the patient coughs, and it is translucent to light. This last is the best and surest test of the presence of a hydrocele. It is not easy to test translucency single-handed, and the examination is much simplified if an assistant is at hand to hold the light in the desired position. The best illumination to use is an electric light, because it can be held sufficiently near the scrotum without causing the patient discomfort. The light is held on the side of the scrotum opposite to the surgeon, who grasps the hydrocele with one hand, at the same time making the skin over it tense, while the other is applied so that its ulnar border lies along the anterior surface of the scrotum, and the hand itself cuts off all rays of light except those transmitted through the hydrocele. A very good way of ensuring that all extraneous rays of light are cut off is to use a hollow roll of paper, one end of which is applied to the scrotum while the other is held to the examining eye. If there is translucency the swelling is seen to have a pinkish colour, while the position of the testicle is shown by a dark spot, which lies in an ordinary case on the posterior surface of the swelling.

It has been said that the presence of translucency is the surest test of the presence of a hydrocele, but it is not absolutely infallible. For instance, a hydatid cyst of the testis or an inguinal hernia in an infant may either of them be translucent, while on the other hand a hydrocele with very thick walls, one whose contained fluid is mixed with blood, or an old hydrocele containing deposits of cholesterin crystals, may be quite impervious to light.

It might be thought that in view of the very definite nature of the signs presented by a vaginal hydrocele, the diagnosis should be an easy matter. This is not the case. As a matter of fact there are few conditions in surgery which call for greater care in the matter of differential diagnosis. A brief consideration of the affections which may be confused with a vaginal hydrocele will, therefore, not be out of place.

The condition of all others most likely to lead to confusion is an ordinary inguinal hernia. As a matter of fact, a careful examination should preclude all possibility of such an error. A hernia gives an impulse when the patient coughs, because the descent of the diaphragm increases the intra-abdominal pressure, and more intestine is forced into the hernial sac. Very rarely a hydrocele gives such an impulse. This happens when the upper extremity of the distended tunica vaginalis has insinuated itself between the pillars of the external abdominal ring. An ordinary hernia can be completely reduced by manipulation, or may disappear spontaneously when the patient assumes the recumbent position. This never happens in the case of a hydrocele. A hernia has its largest diameter in the upper part of the scrotum; a hydrocele, on the other hand, is larger in its lower half. A hernia gives an impression of being irregular to the examining hand, on account of its contained gut or omentum, whereas the surface of a hydrocele is uniform. Further, as has already been said, a hydrocele is translucent; the only form of hernia which transmits rays of light being an inguinal hernia in an infant, when the intestine in the hernial sac contains only flatus.

The differential diagnosis is more complicated in those cases in which hernia and hydrocele are present at one and the same time, especially if for any reason the hernia has become an irreducible one. If, however, the hernia can be reduced, and if, after complete reduction, there still remains in the scrotum a swelling which has all the characteristics of a hydrocele, the diagnosis should be simple. When mistakes are make it is due to the tendency, and it is a very human one, to attempt to attribute all the phenomena to one cause. In nine cases out of ten, or perhaps in a greater proportion still, one is right in going on the hypothesis that there is but one *fons et origo* for all the phenomena found. But one has now and then to recognise the fact that a patient may be suffering at the same time from two entirely different conditions; and this is a case in point. In such a case, if the diagnosis cannot be cleared up by the ordinary method of investigation, an exploratory operation should be undertaken. The only other condition likely to be mistaken for a hydrocele is a hæmatocele. In the latter case the swelling has come up suddenly, often after a definite injury, and is not at all translucent. The diagnosis between hydrocele and disease of the testis itself is only difficult when these co-exist; but a vaginal hydrocele secondary to testicular disease is usually a small one, and does not often mask the very definite changes found in the testis and cord.

